# A flexible nanobrush pad for the chemical mechanical planarization of Cu/ultra-low-к materials

**DOI:** 10.1186/1556-276X-7-603

**Published:** 2012-10-30

**Authors:** Guiquan Han, Yuhong Liu, Xinchun Lu, Jianbin Luo

**Affiliations:** 1 State Key Laboratory of Tribology, Tsinghua University, Beijing 100084, China

**Keywords:** Flexible nanobrush pad, Polishing pad, Chemical mechanical planarization, Cu/ultra-low-к integration

## Abstract

A new idea of polishing pad called flexible nanobrush pad (FNP) has been proposed for the low down pressure chemical mechanical planarization (CMP) process of Cu/ultra-low-к materials. The FNP was designed with a surface layer of flexible brush-like nanofibers which can ‘actively’ carry nanoscale abrasives in slurry independent of the down pressure. Better planarization performances including high material removal rate, good planarization, good polishing uniformity, and low defectivity are expected in the CMP process under the low down pressure with such kind of pad. The FNP can be made by template-assisted replication or template-based synthesis methods, which will be driven by the development of the preparation technologies for ordered nanostructure arrays. The present work would potentially provide a new solution for the Cu/ultra-low-к CMP process.

## Background

With the development of semiconductor industry, the feature size of device is scaling down, and the density of integrated circuit (IC) is continuously increasing, as well as the wafer sizes. As a result, the fabrication techniques are facing new challenges. For instance, the conventional silica is replaced by ultra-low-к materials integrating with copper (Cu) for reduction of the dielectric permittivity [1]. Chemical mechanical planarization (CMP), which is thought as the only one that can offer excellent local and global planarization at the same time, has become one of the most important fabrication technologies adopted by the semiconductor industry [2]. However, due to low density, poor mechanical strength, and deficient adhesion properties, ultra-low-к dielectrics may be damaged by stresses applied during the conventional CMP [3]. The pace of incorporating advanced ultra-low-к materials has been slowing down as compared to the original projections [1,4]. One solution is to reduce down pressure in the CMP process [5]. However, the low down pressure leads to a low material removal rate (MRR) in the CMP process with conventional polishing slurries and pads [6]. Therefore, it is an urgent problem to be solved for the planarization of wafers by CMP under the low down pressure.

Numerous attempts have been made to meet the new Cu planarization requirements due to the use of fragile ultra-low-к materials in the near future. Most of them are focused on slurries [7-11] and the derivative technologies of CMP such as electrochemical mechanical planarization [12-15] and electrochemical mechanical deposition [16-18]. As we know, the polishing pad is one of the most important consumables and plays a critical role in CMP. However, up to now, very few researches have been done on the polishing pad for the low down pressure CMP process of Cu/ultra-low-к materials. Kasai et al. [19] reported a next generation pad with soft materials and smaller pore size (from 2 to 10 μm) to reduce scratch defects. Sung et al. [20] pointed out that ‘dry spots’ of polishing could be caused by this soft pad with smaller pores, and they designed a black pore-free pad with microscope graphite particles impregnated in a polyurethane matrix. Some new kinds of pads potentially used for the low down pressure CMP process have also been reported, such as the eSQ pad [21] based on a compression compliance mechanism and the low-shear surface-engineered pad [22] using ‘pad engineering’ technologies. Most of them are conceptual, confidential, and not fully developed.

Polishing pads [23-30] with free fibers on the surface have been widely studied due to their numerous advantages in the CMP process under normal down pressure, i.e., from 2 to 8 psi. However, the polishing pad with ordered nanofiber arrays on the surface has been seldom reported so far, and its CMP performances under the low down pressure (less than 1 psi or even 0.5 psi) are yet unknown. Previous simulation and experimental works in our group have already indicated that the interaction between abrasive particles and wafer surface has important effects on the CMP performances [31-38]. In the present work, a new idea of polishing pad called flexible nanobrush pad (FNP) has been proposed. A large number of flexible brush-like nanofibers which are supposed to be useful for the low down pressure CMP process of Cu/ultra-low-к materials will be made in the surface layer of the pad. The material removal and planarization mechanisms of the FNP, as well as the possible implementations, have been discussed.

## Presentation of the hypothesis

Polishing pad is one of the most important components in CMP, while it is also one of the most poorly understood components. Pad structures and materials have changed little in the past few decades since the CMP technology was used in the semiconductor industry; nevertheless, the evolution is arrived at empirically for the most part [39].

**Figure 1:**
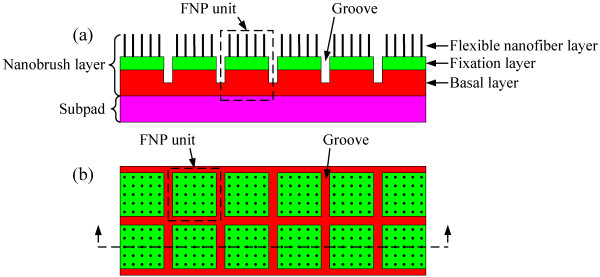
**Schematic diagram of flexible nanobrush pad.** (**a**) Side view and (**b**) top view.

A schematic diagram of the FNP is shown in Figure 1. The FNP includes two layers, i.e., a nanobrush layer and a subpad. The nanobrush layer consists of a flexible nanofiber layer, a fixation layer, and a basal layer. Similar to the conventional porous polishing pad, the FNP can optionally have macro-textures such as grooves in the surface for the purpose of better slurry delivery. The basal layer, made of hard materials, provides a support for the flexible nanofiber layer and the fixation layer. It also maintains a high enough stiffness for the surface layer of the FNP, which is very essential to achieve the good planarization. The subpad made of soft materials enables the FNP to conform to wafer surface flatness variation and achieve good polishing uniformity. The top-hard and bottom-soft structures, inheriting from the conventional stacked pad, can achieve both good planarization and polishing uniformity [40]. The biggest difference between the FNP and the conventional pad is that there are a large number of flexible brush-like nanofibers (i.e., flexible nanobrush) in the surface layer of the FNP rather than micropores or microfibers. The functions of the flexible nanobrush will be discussed in particular below.

**Figure 2:**
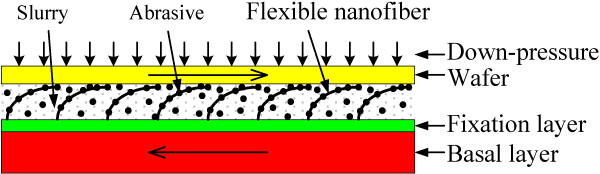
Material removal mechanism on mechanical aspects in CMP using flexible nanobrush pad.

The most serious challenge under low down pressure for conventional CMP is its low MRR. How is MRR raised under the low down pressure using the FNP? In fact, it has been believed that the CMP system is mechanically limited at low down pressures [41-43]. Meanwhile, the mechanical removal rate depends on how many abrasives on the pad are pressed against the wafer, with the indentation depth of the abrasives being proportional to the applied pressure [44,45]. Therefore, the key to improve the MRR under the low down pressure is to increase the number of abrasives in contact with the wafer and the contact area between the pad and the wafer. From this point, the FNP has been designed. Material removal mechanism on mechanical aspects in CMP by the FNP is shown in Figure 2. By adjusting the types and properties of the nanofibers and slurry compositions, it is believed that nanoscale abrasives in slurry can be ‘actively’ carried by the nanofibers during polishing. The large number of nanofibers can absorb large quantities of abrasives. As a result, the contact frequency between the pad and the wafer can be greatly increased, as well as the contact area. Hence, the MRR can be enhanced to a great content. The abrasive-carrying capability of the FNP is independent of the down pressure, and meanwhile, only a small force is needed to keep the contact between the nanobrush and the wafer. Therefore, a high MRR can also be expected under the low down pressure.

Furthermore, because of the existence of flexible nanofiber layer, the contact between the pad and the wafer will become more uniform; thus, the potential contact ‘hot spots’ [21] can be eliminated. Slurry can be sucked to the contact area along the nanofibers by capillary force, so ‘dry spots’ [20] of polishing can also be avoided during the CMP process. Therefore, a better wafer surface with reduced scratches can be anticipated. In addition, the residual abrasives and polishing byproducts such as pad debris could be cleaned out by just using ultrasound, rinse, or other means. Hence, it becomes easier to maintain the FNP performance without diamond conditioning used by the conventional porous pad.

## Testing the hypothesis

**Figure 3:**
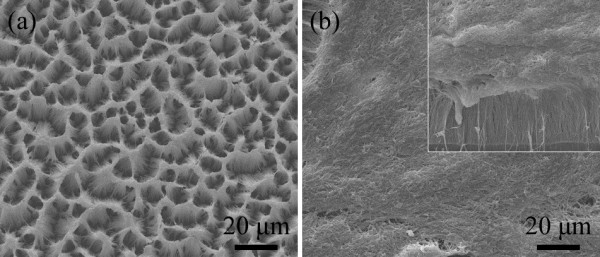
**Scanning electron microscope images of two FNPs prototyped by a template-assisted synthesis.** (**a**) The surface of one FNP; (**b**) the surface of another FNP and the cross-section of its nanofiber layer (inset).

The crucial difficulty in producing the FNP is how to fabricate large-area flexible ordered nanofiber arrays. The diameter of the conventional porous pad has been up to 1 m. However, it is very difficult to fabricate such a large nanofiber array with present existing techniques. Fortunately, there still have a few approaches to fabricating small-area ordered nanofiber arrays, such as template-based replication methods [46-49] and template-assisted synthesis [50-55], despite these technologies are yet immature. On the other hand, considering the grooves at the pad surface, we can use many small-area arrays (i.e., the small FNP unit as shown in Figure 1) to make up a large-area FNP. We can improve the existing polishing tool to test and optimize the performance of the FNP and develop other matching technologies such as slurries, process parameters, and pad conditioning technologies. As shown in Figure 3, two small area FNP units have already been prototyped by a simple template-assisted synthesis using anodic aluminum oxide membrane and thermoplastic polyurethane solution. In the FNP, as shown in Figure 3a, the nanofibers are clustered and tend to be perpendicular to the pad surface. In another FNP, as shown in Figure 3b, the up ends of the nanofibers are fallen, but the roots (as shown in the inset of Figure 3b) are perpendicular to the pad surface. The preparation, characterization, and evaluation of the prototypal FNP will be detailedly discussed in our future work.

## Implications of the hypothesis

As semiconductor technology develops and new materials are introduced for more advanced ICs, novel consumables of CMP must be developed to meet these new requirements. The work puts forward a flexible nanobrush technique for the polishing pad used in the low down pressure CMP process of Cu/ultra-low-к materials. Better polishing performances including high material removal rate, good planarization, good polishing uniformity, and low defectivity are expected to be achieved with such kind of pad. The FNP can be prototyped by template-assisted replication or template-based synthesis methods. It is expected that the work would potentially provide a new solution for the Cu/ultra-low-к CMP process. It is also expected that the work can be driven by the development of the preparation technologies for ordered nanostructure arrays.

## Abbreviations

CMP: chemical mechanical planarization; FNP: flexible nanobrush pad; MRR: material removal rate; IC: integrated circuit.

## Competing interests

Patent concerning flexible nanobrush pad and manufacturing methods thereof is pending (China patent 201010217079.5, 2010).

## Authors' contributions

GH, YL, and JL conceived of the study and participated in its design and coordination. GH drafted the manuscript. YL, JL, and XL were involved in revising the manuscript. All authors read and approved the final manuscript.

## Authors' information

GH is a Ph.D. candidate and engages in novel chemical mechanical planarization research. YL is a doctor, assistant professor, and an expert in the field of nanostructures and nanotechnology. XL is a doctor, professor, and an expert in equipment and technology of CMP. JL is a doctor, professor, an academician of Chinese Academy of Sciences (CAS), and director of State Key Laboratory of Tribology (SKLT) and specializes in tribology and nanomanufacturing.
